# Impact of captivity and natural habitats on gut microbiome in *Epinephelus akaara* across seasons

**DOI:** 10.1186/s12866-024-03398-y

**Published:** 2024-07-03

**Authors:** Hang Sun, Fangyi Chen, Wenbin Zheng, Yixin Huang, Hui Peng, Hua Hao, Ke-Jian Wang

**Affiliations:** 1 State Key Laboratory of Marine Environmental Science, College of Ocean and Earth Sciences, Xiamen University, Xiamen, Fujian China; 2 State-Province Joint Engineering Laboratory of Marine Bioproducts and Technology, College of Ocean and Earth Sciences, Xiamen University, Xiamen, Fujian China; 3 Fujian Innovation Research Institute for Marine Biological Antimicrobial Peptide Industrial Technology, College of Ocean and Earth Sciences, Xiamen University, Xiamen, Fujian China

**Keywords:** *Epinephelus akaara*, Gut microbiota distribution, Diversity, Season, Captive and wild

## Abstract

**Background:**

The gut microbiota significantly influences the health and growth of red-spotted grouper (*Epinephelus akaara*), a well-known commercial marine fish from Fujian Province in southern China. However, variations in survival strategies and seasons can impact the stability of gut microbiota data, rendering it inaccurate in reflecting the state of gut microbiota. Which impedes the effective enhancement of aquaculture health through a nuanced understanding of gut microbiota. Inspired by this, we conducted a comprehensive analysis of the gut microbiota of wild and captive *E. akaara* in four seasons.

**Results:**

Seventy-two *E. akaara* samples were collected from wild and captive populations in Dongshan city, during four different seasons*.* Four sections of the gut were collected to obtain comprehensive information on the gut microbial composition and sequenced using 16S rRNA next-generation Illumina MiSeq. We observed the highest gut microbial diversity in both captive and wild *E. akaara* during the winter season, and identified strong correlations with water temperature using Mantel analysis. Compared to wild *E. akaara*, we found a more complex microbial network in captive *E. akaara,* as evidenced by increased abundance of *Bacillaceae*, *Moraxellaceae* and *Enterobacteriaceae*. In contrast, *Vibrionaceae*, *Clostridiaceae*, *Flavobacteriaceae* and *Rhodobacteraceae* were found to be more active in wild *E. akaara.* However, some core microorganisms, such as *Firmicutes* and *Photobacterium*, showed similar distribution patterns in both wild and captive groups. Moreover, we found the common community composition and distribution characteristics of top 10 core microbes from foregut to hindgut in *E. akaara*.

**Conclusions:**

Collectively, the study provides relatively more comprehensive description of the gut microbiota in *E. akaara,* taking into account survival strategies and temporal dimensions, which yields valuable insights into the gut microbiota of *E. akaara* and provides a valuable reference to its aquaculture.

**Supplementary Information:**

The online version contains supplementary material available at 10.1186/s12866-024-03398-y.

## Introduction

Gut microbiota is intricately linked to the health and disease of a variety of species [1]. These tight relationships contribute to host development, behavior, metabolism, immunity and various other processes, including speciation [2–8]. The interaction between microbes and host is bidirectional and persists over the hosts’ lifetime via established exposure rotes, notably diet, social interactions, biogeography, seasonality, maternal sources [9–14]. Therefore, understanding the spatial and temporal processes that shape gut microbial communities is an important unresolved objective [14]. Perturbations to these relationships can cause variations in gut microbiota, even within individuals of the same species. For instance, the gut microbial composition and diversity in great apes [15] and deer mice [16] exhibit alterations influenced by the surrounding environment in both wild and captibity. Moreover, the host microbial communities are subjected to seasonal variations. The microbial communities in wildlife populations, such as mice, Galápagos vampire finch, Zebrafish, red squirrels [13, 14, 17, 18], display variations influenced by seasonal factors. However, the characteristic of gut microbiota and the spatial and temporal interactions of the microbiota in captive and wild animal populations remain poorly understood.

Fish, account for over half of all vertebrate species, making them an integral part of biodiversity, they are also economically important and serve as a crucial source of animal protein for humans [19]. With an increasing global population and limited fisheries resources, aquaculture is now among the world's most rapidly expanding animal production sectors [20]. The gut microbiota plays a crucial role in fish health [21]. Understanding the distinctions in fish gut microbiota between captive and wild environments aross four seasons provides valuable insights into the spatial and temporal factors influencing inter-individual variations, thereby significantly contributing to effective disease control in aquaculture [14]. Furthermore, the gut microbiota of aquatic animals may exhibit variations in response to alterations in the marine environment [22] or aquaculture environments [23], which can be seasonal and accompanied by fluctuations in water temperature [24, 25]. For example, gut microbiota of captive tilapia (*Oreochromis niloticus* × *Oreochromis aureus*) revealed substantial seasonal discrepancies, particularly during winter in contrast to the other seasons [25]. Seasonal fluctuations contribute significantly to the variability in the gut microbiota of European abalone held in captivity (*Haliotis tuberculata*), leading to changes in community structure [26]. Seasonal animal migration of wild grey mullet (*Mugil cephalus*) leads to variable microbial communities, especially in *Synechococcaceae* [27]. On the other hand, numerous studies have extensively documented significant divergences in the gut microbial communities of marine animals between the wild and those held in captivity. Comparative studies have been conducted on several species, including *Salmo salar, Gadus morhua*, *Paralichthys adspersus*, *Chelonia mydas* and *Poecilia reticulata* [28–31]. Although the impacts of seasonality and divergence between wild and captive fish on these processes have been reported separately, the interactions between the centralized microbiota and spatial–temporal factors in wild and captive fish remain largely unknown.

The red-spotted grouper (*E. akaara*), stands as one of Asia's most popular and economically significant marine fish species, as it is farmed on a large scale for domestic consumption and export [32, 33]. Fujian boasts China's second-longest coastline, and *E. akaara* ranks as the second most productive marine commercial fish species in the province [34]. In this study, *E. akaara* was selected and a variety of environmental parameters, such as season, location and water were monitored on both wild and capture red-spotted grouper over a period of one year. As in other vertebrates, the distribution and function of the fish gut microbiota exhibit significant variations across gut sites, which may be influenced by host genetics and selective pressures associated with functions such as nutrient absorption and immunity. Consequently, studies on fish gut microbiota are affected by the diversity of fish gut segmentation methods and require enhanced sampling and experimental methodologies [8].

Considering the impact of different segmentation methods on the study of fish gut microbiota and the intrinsic variability of microbial composition and function in different gut sites, we sampled the foregut, midgut, hindgut, and content of *E. akaara*, following the methodology of a previous study [35]. This was done to obtain relatively complete data on the gut microbiota of *E. akaara* and to elucidate the distribution of microorganisms in different gut sites. Based on the collected data, we analyzed the significance of captive and natural habitats in shaping the gut microbial community of *E. akaara* during four seasons (spring, summer, autumn, and winter) in southeastern China. Which provide a comprehensive view of the red-spotted grouper and support a model for understanding the interactions between microbiota, temporal factors and ecological conditions.

## Results

### Diversity of the gut microbiota of wild and captive *E. akaara* during four seasons

**Fig. 1:**
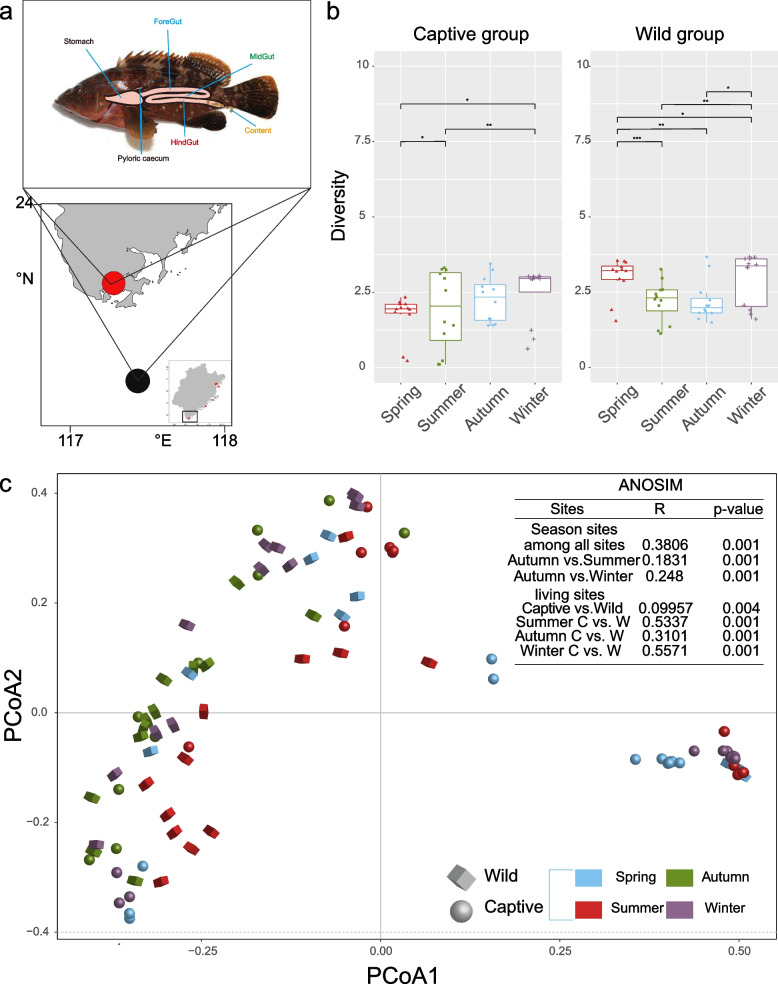
Diversity of gut microbiota between captive and wild *E. akaara* during four seasons. **a** Spatial structure of the digestive tract of the *Epinephelus akaara* and our four sampling sites (foregut, midgut, hindgut and content). Study area and the sampling sites in Dongshan City of Fujian province (the captive *E. akaara* collected from the red site while the wild collected from the black site). **b** ɑ-Diversity comparison based on the Shannon diversity index in each of the parts using ANOVA to determine significant differences (*P < 0.05, ** *P* < 0.01, *** *P* < 0.005, ns *P* > 0.05). In data shown as a combination of dot plots and box plots. **c** Principal coordinate analysis plot generated using OTU metrics based on the Bray–Curtis dissimilarities. Each point represents a sample. Differences were assessed by ANOSIM and significance was established at *P* < 0.05. An R-value close to ‘1’ suggests dissimilarity between groups, whereas an R-value close to ‘0’ suggests an even distribution of high and low ranks within and between groups

A total of 4971 OTUs and 6,926,198 sequences (mean: 57,718, min: 51,409, max: 170,617, SD =  ± 24,717.63) were present in all *E. akaara*, with an average of 300 ± 220 unique OTUs per individual. A total of 72 individuals were captured over the four seasons (*n* = 36 captive born; *n* = 36 wild born). To gain a deeper understanding of the gut microbial communities in *E. akaara*, we collected four gut parts from 72 individuals (Fig. 1a, foregut, midgut, hindgut and contents, Table S1). These gut sections were collectively analyzed to assess the composition and distribution characteristics of microbial communities.

First, to evaluate variations in community structure and diversity across distinct seasons, we conducted α and β-diversity analyses. The analysis of taxon evenness, as indicated by Shannon indices, revealed differences in gut microbial diversity between captive and wild *E. akaara* (Fig. 1b). The Shannon diversity index of both captive and wild *E. akaara* peaked in winter. Specifically, diversity in the captive group was significantly higher in winter than in spring and summer (*P* < 0.05). Similarly, in the wild group, the level of diversity was significantly higher in winter than in summer and autumn (*P* < 0.05). To further evaluate β diversity, we conducted principal coordinate analysis (PCoA) to visualize the variations in taxon composition across seasons. The PCoA score plot (Fig. 1c) showed a significant separation between sites across all seasons, which was supported by the ANOSIM results (*R* = 0.3806, *P* = 0.001). The discrete distributions of captive and wild *E. akaara* were significant changed in summer and winter, as evidenced by the significant differences observed in summer (*R* = 0.5337, *P* = 0.001) and winter (*R* = 0.5571, *P* = 0.001). The difference of four seasons was found to be greater between autumn and winter than that of the other seasons (*R* = 0.248, *P* = 0.001). Additionally, seasonal fluctuations exert discernible influences on the microbiota composition across various gut sites of both captive and wild *E. akaara*, with relatively minor effects observed on the contents (Fig. S1). This might suggest that the composition of the microbiome in summer and winter became more variable as a result of seasonal changes.

### Distribution pattern of core microbes in both captive and wild *E. akaara* from spring to winter

**Fig. 2:**
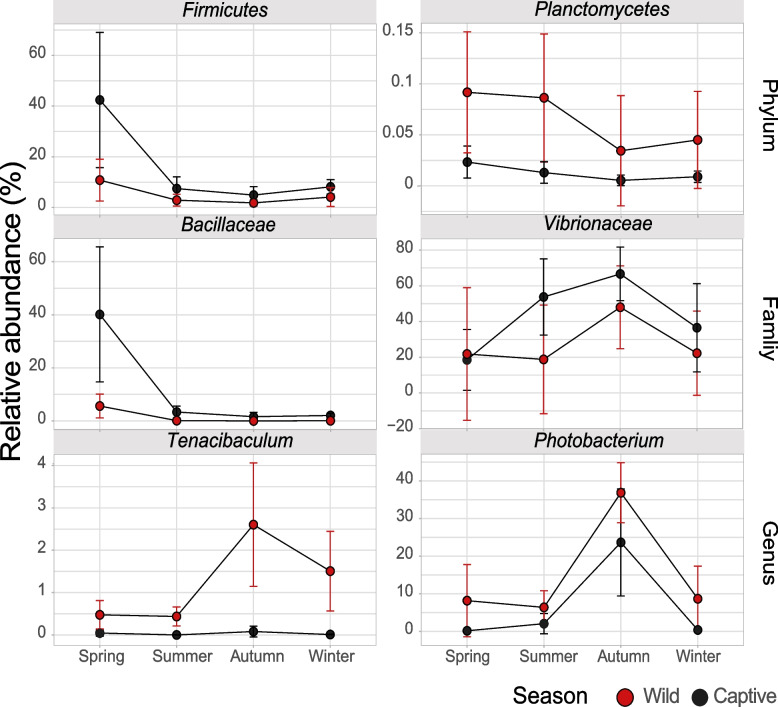
Distribution pattern of core microbes in both captive and wild *E. akaara* from spring to winter. Relative abundance of specificity patterns affected by seasonal variations at phylum, family and genus level

We subsequently analyzed the gut microbial composition in both captive and wild *E. akaara* from spring to winter. Notably, a number of core microbes (core bacteria shared in all samples, > 0.5%) in wild and captive *E. akaara* displayed similar distribution patterns (peak, minimum value or others) across seasons at the phylum, family, and genus levels, and similar fluctuations in abundance were observed throughout all four seasons (Fig. 2). *Firmicutes* and *Planctomycetes* were detected in all fish samples, and their relative abundance peaked in the spring in both the captive and wild groups, followed by decreases in the summer, autumn and winter. At family level, *Bacillaceae* showed the same pattern as *Firmicutes* and *Planctomycetes*, while *Vibrionaceae* displayed the opposite pattern, with an upward trend from spring to autumn, reaching its peak during the autumn season in both captive and wild groups. At genus level, *Tenacibaculum* and *Photobacterium* showed similar trends from spring to winter. Additionally, we also observed some interesting distribution patterns of core microbes that interacted with seasonal variation (Fig. S2). These results suggested that seasonal variation sufficiently affected the microbial community composition of both wild and captive *E. akaara*.

### Water environmental factors could potentially drive shaping the core gut microbial communities of both captive and wild *E. akaara* across four seasons

**Fig. 3:**
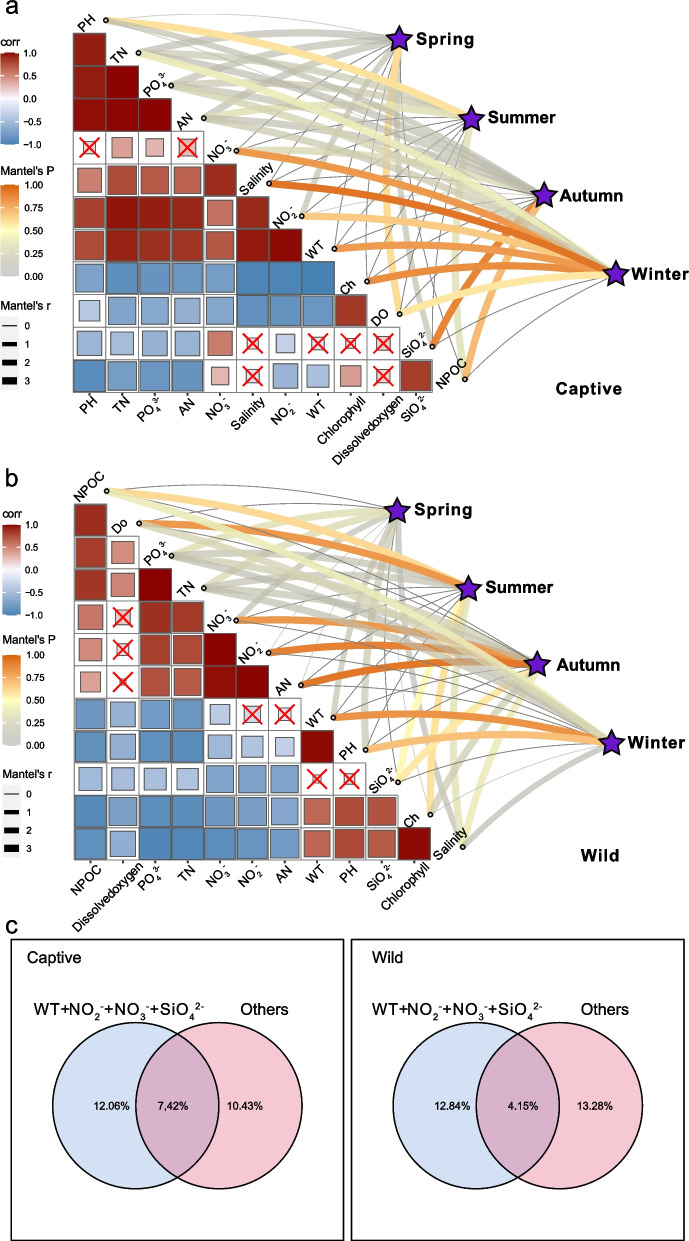
Environmental drivers of the microbial communities between captive and wild *E. akaara* during four seasons (Bray–Curtis distance) and environmental factors were analyzed with Mantel tests. **a** The mantel test between the microbial communities in captive *E. akaara* and environmental factors. **b** The mantel test between the microbial communities in wild *E. akaara* and environmental factors. The edge width corresponds to the R-value, and the edge color denotes the statistical significance. The color gradient indicates Pearson correlation coefficients among the environmental factors indicate no significant correlation at 0.05 level. **c** Variation partitioning analysis (VPA) separating the variation of community structure explained by the RDA model

Waterborne bacterial communities serve as crucial reservoirs of intestinal microbiota. The variations in microbial populations within the aquatic environment across the four seasons are depicted in Fig. S3. However, building upon our prior research, which found significant correlations between environmental factors and changes in the gut microbiota of *E. akaara* across five regions [35], we further investigated how various environmental factors influence the microbial communities in both captive and wild *E. akaara* over four seasons (Table S2), without examining the impact of aquatic environmental microbes on gut microbiota. In the captive *E. akaara* group, the spring microbial community demonstrated a noteworthy correlation with DO (*P* < 0.01) (Fig. 3a), and the summer group demonstrated a notable correlation with PH (*P* < 0.01). The autumn group demonstrated a significant correlation with SiO_4_^2−^ and NPOC. The winter group demonstrated a noteworthy correlation with DO, NPOC, chlorophyll, WT, NO_3_^−^, NO_2_^−^ and salinity. However, in the wild *E. akaara* group, the summer microbial community demonstrated a noteworthy correlation with NPOC, SiO_4_^2−^, chlorophyll and DO (*P* < 0.01) (Fig. 3b). The autumn group demonstrated a notable correlation with SiO_4_^2−^, NO_3_^−^, NO_2_^−^ and AN. The winter group exhibited a significant correlation with PH and WT. Variation partitioning analysis (VPA) outcomes reveal that WT, NO_2_^−^, NO_3_^−^, and SiO_4_^2−^ collectively elucidate up to 12.06% of the bacterial community structure variance in cultured group across four seasons (Fig. 3c). Additionally, these environmental factors contribute to an explanation degree of 12.84% concerning the bacterial community structure in wild group across the same four seasons. These findings further underscore the substantial role of aquatic environmental factors in influencing the disparities in microbial communities between captive and wild *E. akaara* across all four seasons.

### The microbial community composition of captive *E. akaara* was more complexity than that of wild *E. akaara*

**Fig. 4:**
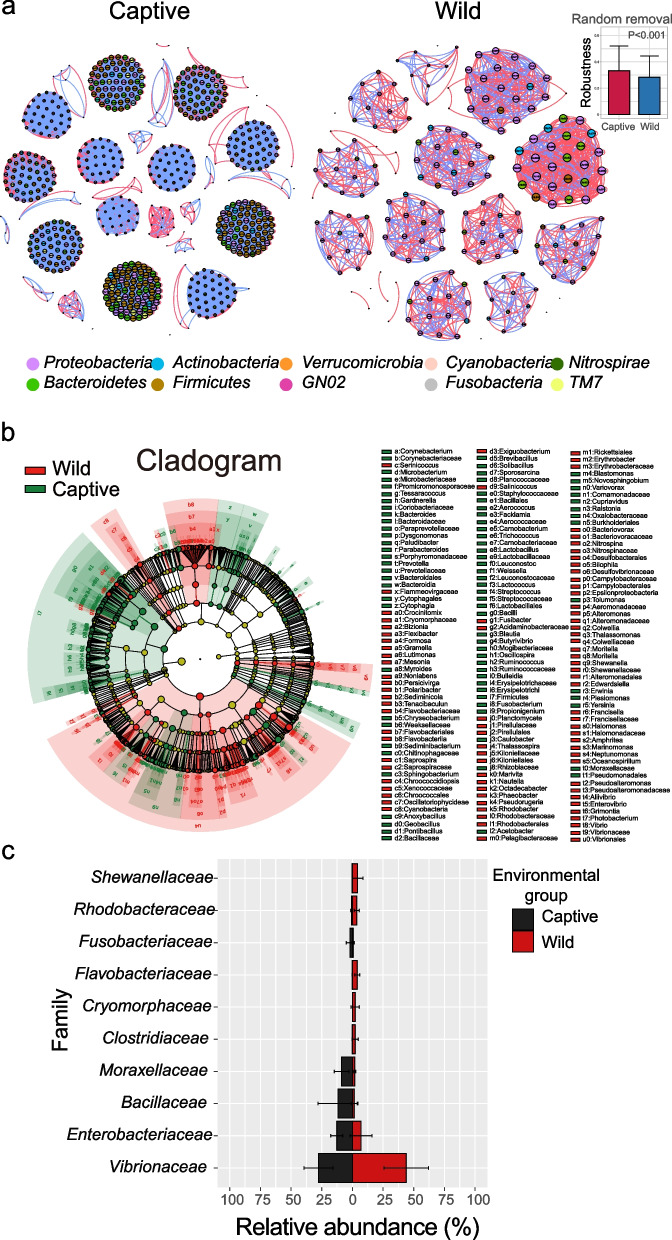
The composition of microbial communities in captive showed more complexity than wild *E. akaara.* **a** The networks of co-occurring bacterial OTUs in *E. akaara* between captive and wild group, based on correlation analysis. The co-occurrence networks are colored by phylum. A red edge indicates a positive interaction between two individual nodes, while a blue edge indicates a negative interaction. And the robustness measured as the proportion of taxa remained with 50% of the taxa randomly removed from each of the co-occurrence networks. **b** Cladogram generated from linear discriminant analysis (LDA) effect size (LEfSe) showing the relationship between taxon (the levels represent, from the inner to outer rings, phylum, class, order, family, and genus) between captive and wild *E. akaara*. **c** Side-by-side comparison of the mean relative abundance of genus which made up at least > 1% of the total gut microbiome community, within at least one individual, between captive and wild *E. akaara*

As biological data sets continue to grow in size and scope, scientists have increasingly adopted novel techniques, including network analysis, to comprehend the intricacies of large-scale biological systems [23, 36]. Here, utilizing robust and statistically significant correlations, we delved into the bacterial co-occurrence patterns within *E. akaara* in both captive and wild groups over four seasons using network analysis [23, 37, 38]. Overall, the ecological networks of these two groups were markedly different. The captive group exhibited markedly greater network complexity in contrast to the wild group, as supported by the substantial presence of co-occurring taxa (OTU) (Fig. 4a). Furthermore, the random removal robustness analysis provided additional evidence supporting the complexity and stability of the co-occurrence network within the cultured group (*P* < 0.001). Within these two networks, the prevalence of positive correlations significantly exceeded that of negative correlations, with over 61% of correlations being positive (Fig. 4a and Table S3). Overall, significant disparities in the topological properties across these empirical networks indicated marked differences the composition of bacterial communities between captive and wild *E. akaara*. The correlations within the bacterial community of captive E. akaara were found to be more robust when compared to those of the wild E. akaara, as indicated by the quantities of nodes and edges (Table S3). Furthermore, the values of average path length (APL) and average clustering coefficient (avgCC) showed a similarity between captive and wild E. akaara, indicating that both empirical networks exhibited prominent “small world” modularity. Additional structural analysis unveiled a deterministic pattern of co-occurrence in the bacterial networks at the family level. Remarkably, the bacterial OTUs in the predominant families, including *Proteobacteria*, *Actinobacteria*, *Verrucomicrobia*, *Cyanobacteria*, *Bacteroidetes* and *Firmicutes*, exhibited a higher tendency to co-occur compared to other families in the networks. Moreover, the co-occurrence networks observed across different gut parts in both captive and wild group across four seasons provide additional evidence of the influence of seasonal dynamics on symbiotic interactions and taxonomic attributes, highlighting disparities between captive and natural environments as well as among different gut parts (Fig. S4-S5). For example, in spring, the co-occurrence networks in the foregut exhibited more complex and stable features in both captive and wild habitats. However, in autumn, microbial symbiotic networks in the foregut, midgut, hindgut, and contents were less complex in captive habitats compared to wild habitats.

The LEfSe analysis results revealed notable alterations in the core microbial composition between captive and wild groups (Fig. 4b). The captive group was enriched in 82 key species, such as *Bacilli*, *Moraxellaceae*, and *Fusobacterium*, whereas the wild group was enriched in 86 key species, including *Vibrio*, *Shewanella*, and *Rhodobacter*. Furthermore, based on the LEfSe analysis results, we further observed significant differences in dominant phyla, families, and genera when comparing captive and wild individuals (Fig. 4c, Fig. S6 and Fig. S7). At the family level, *Vibrionaceae*, *Enterobacteriaceae*, *Bacillaceae* and *Moraxellaceae* dominated the gut microbiota of *E. akaara. Enterobacteriaceae*, *Bacillaceae* and *Moraxellaceae* were more abundant in captive *E. akaara*, while *Vibrionaceae* was more prevalent in wild *E. akaara* (Fig. 4c). These results suggested that the decreased network complexity in captive *E. akaara* might be due to the high abundance of *Vibrionaceae*.

### Common distribution patterns of microbial communities across distinct gut fragments in *E. akaara*

**Fig. 5:**
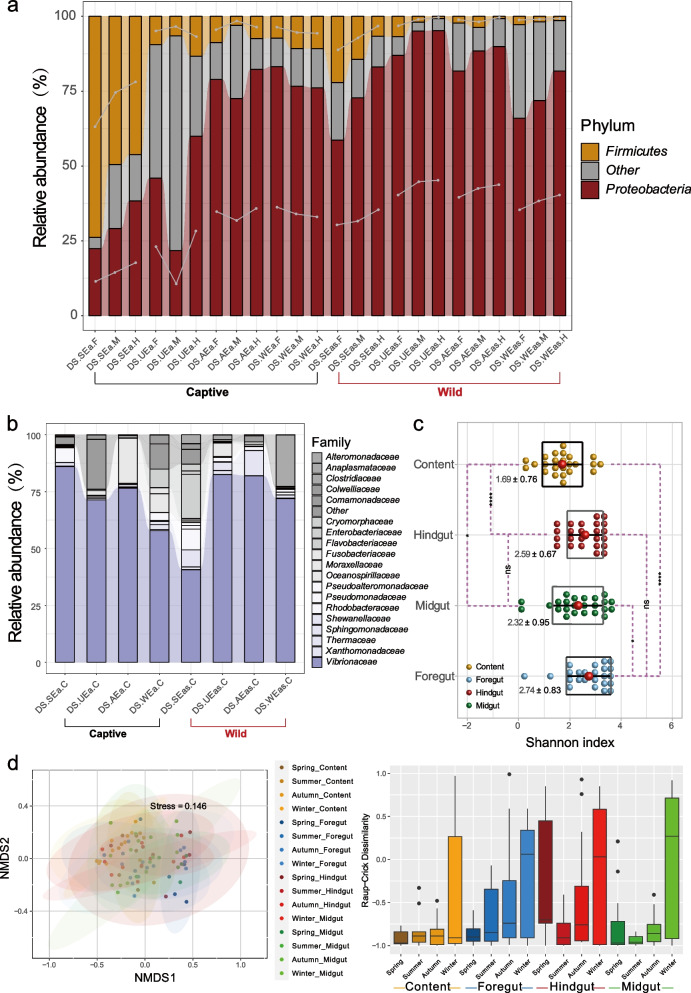
Common distribution characteristics of microbial communities in different gut fragments of *E. akaara.* **a** The relative abundance of the microbial communities at the phylum level found in different gut compartments. F, foregut. M, midgut. H, hindgut. C, content. SEa, Spring captive *E. akaara.* UEa, Summer captive *E. akaara.* AEa, Autumn captive *E. akaara.* WEa, Winter captive *E. akaara.* SEas, Spring wild *E. akaara.* UEas, Summer wild *E. akaara.* AEas, Autumn wild *E. akaara.* WEas, Winter wild *E. akaara.* Each group represents 9 fish individuals with a parallel sample mixed by three individuals. Only *Proteobacteria* and *Firmicutes* of different gut parts are plotted. **b** The relative abundance of the microbial communities at the family level found in content. Only the dominant microbial family with top 20 of the content are plotted. **c** ɑ-Diversity comparison based on the Shannon diversity index in each of the parts using ANOVA to determine significant differences (** *P* < 0.01, **** *P* < 0.001, ns *P* > 0.05) In data shown as a combination of dot plots and box plots (*n* = 108 fish individuals), with the center red point indicates the mean value in the corresponding group and the data are expressed as the means ± SD. **d** null model based on the Raup-Crick index to elucidate the relative significance of deterministic versus stochastic processes in community assembly. Non-metric multidimensional scaling (NMDS) analysis and intra-group box plot based on the Raup-Crick dissimilarity index

Following an investigation of various time scales (different seasons) and different culture environments, we expected to identify the distributional characteristics of individual gut microbiota in *E. akaara*. As the microbial communities were found to colonize equally along the different gut parts [35], we observed interesting distribution patterns from the foregut to the hindgut. The amplicon sequencing of gut microbial communities in *E. akaara* unveiled noteworthy disparities in the distribution patterns among distinct gut segments. Specifically, we observed an increasing trend in *Proteobacteria* and *Vibrionaceae,* and a decreasing trend in *Firmicutes* abundance from the foregut to the hindgut (Fig. 5a). And *Photobacterium*, *Vibrio*, and *Aliivibrio* make a significant contributions to the overall abundance of *Vibrionaceae* (Fig. S8). Furthermore, our investigation revealed that *Proteobacteria* and *Vibrionaceae* comprised the predominant taxa in the content. (Fig. 5b and Fig. S9). The diversity within the content exhibited a notably lower level when compared to the other four sections of the gut, as indicated by the Shannon index (Fig. 5c), with the foregut displaying the highest diversity. Furthermore, we employed a null model based on the Raup-Crick index to elucidate the relative significance of deterministic versus stochastic processes in community assembly [39]. The finding reveals that beta diversity, as indicated by β_RC_, exhibits heightened community similarity among intestinal slices during spring, summer, and autumn, with deterministic processes predominantly driving community assembly. Conversely, in winter, community similarity within the foregut, midgut, and hindgut registers lower levels, indicating stochastic processes as the predominant force shaping community assembly (Fig. 5d).

**Fig. 6:**
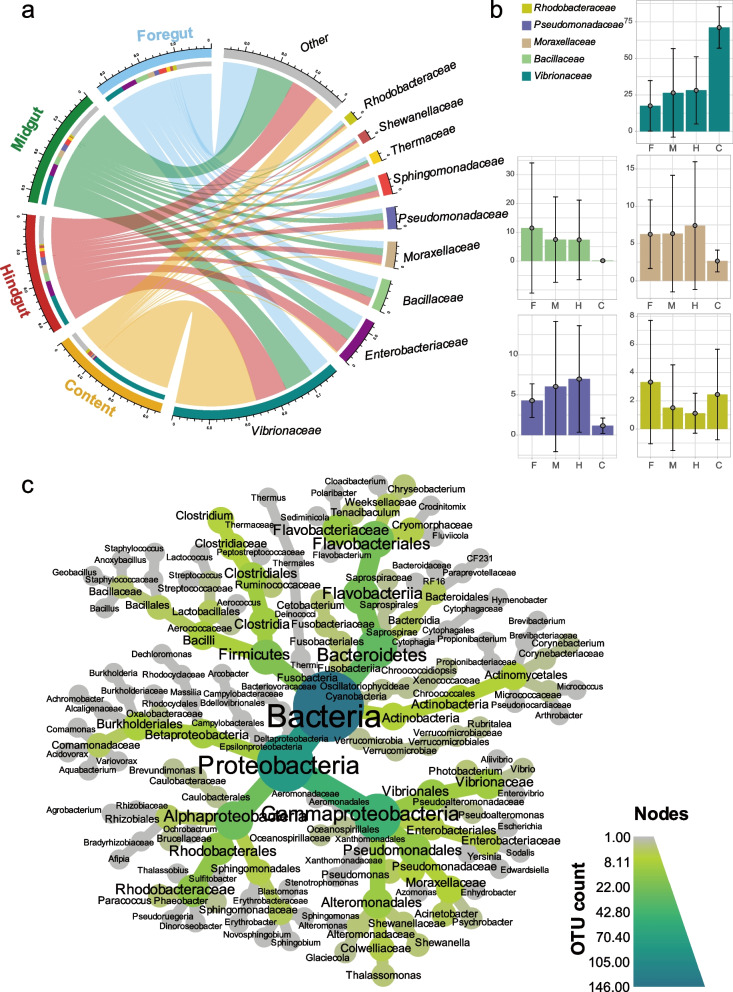
Common distribution characteristics and composition of microbial communities in different gut fragments of *E. akaara.* **a** Relative abundance of the top 10 family in samples of different gut compartments (foregut, midgut and hindgut). **b** Relative abundance of common distribution in different gut fragments at family level. F, foregut. M, midgut. H, hindgut. C, content. **c** The species classification tree displayed the mean proportion of bacterial components. Nodes represent each taxonomic rank from kingdom (bacteria, center) to genus (tips of each branch). Node and edge (branch) width indicates the mean proportion of that taxon in samples belonging to that group. Size of nodes corresponds to the number of taxa and color intensity corresponds to proportions relative to bacterial samples overall. Only genus detected at ≥ 0.03 mean proportion are displayed

To further investigate the taxonomic, compositional and distributional characteristics of the gut microbiota of *E. akaara* for changes in microbial abundance including survival strategies and temporal dimensions, we visualized the species taxonomic tree and compared microbial abundance in different gut parts at family levels (Fig. 6) The top ten dominant families in *E. akaara* were *Vibrionaceae*, *Enterobacteriaceae*, *Bacillaceae*, *Moraxellaceae*, *Pseudomonadaceae*, *Sphingomonadaceae*, *Thermaceae*, *Clostridiaceae* and *Comamonadaceae*, with *Vibrionaceae* being the most abundance at all gut parts (Fig. 6a). The distribution characteristics of the gut microbiota in *E. akaara* were then considered together at the family level with all datasets (Fig. 6b). We observed an increasing trend of *Vibrionaceae*, *Moraxellaceae* and *Pseudomonadaceae* from the foregut to the hindgut, whereas a decreasing trend in *Bacillaceae* and *Rhodobacteraceae*. The species taxonomic tree also showed that the cluster characterized by the similar bacterial communities dominated by various *Vibrionaceae* (mainly *Vibrio* and *Photobacterium*), *Moraxellaceae* (mainly *Acinetobacter* and *Psychrobacter*), *Clostridiaceae* (mainly *Clostridium*) and *Enterobacteriaceae* (Fig. 6c). Moreover,the microbial communities in different gut segments of wild and captive group were performed separately, indicating the divergence of common distribution patterns between wild and captive group, which could be attributed to the varying distributions of core microbiota between captive and wild environments, such as *Enterobacteriaceae* and *Pseudomonadaceae* (Fig S10). These findings reinforce the idea that the gut microbiota in *E. akaara* exhibited common compositional and distributional characteristics that can be determined from investigations of various survival strategies and temporal dimensions.

## Discussion

*E. akaara* is a commercially significant marine fish that is favored and widely consumed by food enthusiasts in southern China, both in the wild and in captivity. Fujian province has a coastline of 3324 km, rich in biological resources and a suitable marine environment for *E. akaara* cultivation throughout the year. Therefore, different regions along the coastline can be selected to culture *E. akaara* to achieve year-round production of both wild and captive *E. akaara* [34]. However, many studies have pointed out that captive and wild *E. akaara* face challenges such as high mortality rates, long growth periods*,* aand declining production in wild *E. akaara* [40]*.* In many vertebrates, such as fish, alterations in the gut microbiota have been documented to exert substantial effects on host health, including metabolism and immune responses during digestion and absorption [5, 41–44]. This indicates that the gut microbiota a pivotal role in fostering fish growth [21] and controlling diseases in fish aquaculture [14]. Here, for the first time, we described the gut microbial communities of free-living *E. akaara* over four seasons, and compared the microbial communities between wild and captive *E. akaara* across seasons to provide a comprehensive perspective on *E. akaara* and contribute to a valuable model for enhancing the understanding of microbiota- timporal and ecological interactions.

The experiment was conducted at a breeding company located off the coast of Dongshan island in Fujian Province, with nearby sea waters subject to specific seasonal variations [26, 45, 46]. The lowest temperatures in subtropical coastal waters occurred in February–April 2018 and January 2018, while the highest temperatures occurred in June- October 2018 (Table S4). These temperature patterns were similar to those observed in temperate coastal waters, possibly due to the proximity of the study area. Numerous studies conducted in temperate coastal regions have shown seasonal succession and structuring in bacterial communities [26, 47–49]. Throughout the year, our results indicated that the gut microbiota community structure of *E. akaara* was influenced by seasonal variations in both captive and wild *E. akaara* (Figs. 1 and 2). In both the wild and captive environments, *E. akaara* exhibited different distribution pattern of α-diversity in each season. In spring, summer and autumn, the diversity levels (Shannon index) of captive *E. akaara* were significantly lower than in winter. In contrast, there was a decreasing trend in the diversity of wild *E. akaara* from winter to autumn (Fig. 1b). In winter, both wild and captive *E. akaara* showed the highest level of gut microbiota diversity compared to the other seasons. However, this finding contrasts with other marine organisms, such as Atlantic salmon (*Atlantic salmon paramyxovirus*) [50], abalone (*Haliotis tuberculate*) [26] and symbitic coral (*Astrangia poculata*) [49], which displayed the highest gut microbiota diversity during the summer months when temperatures were highest. Therefore, we hypothesized that the decrease in diversity during summer might be due to an escalation in the prevalence of specific microbial species within the dominant core, concomitant with a decline in less common microbiota. For instance, the presence of *Vibrio*, *Psychromonas* and *Pseudahrensia* exhibited a positive correlation with elevated water temperatures, leading to higher abundance of core microbes [26, 51]. β-diversity analysis clearly revealed compositional differences, indicating that the microbial community structure of wild and captive *E. akaara* differed significantly in both summer and winter, with relatively high *R* values (summer, *R* = 0.5337, *P* = 0.001, winter, *R* = 0.5571, *P* = 0.001). The gut microbiota of captive and wild *E. akaara* showed some similarity in spring, with a relatively lower *R* value (spring, *R* = 0.2183, *P* = 0.01). This result highlights the substantial differences in microbiome composition between the wild and captive groups across all four seasons.

Notably, we assessed the relative abundances of the top 10 phyla, top 20 families and top 20 genus. These highly abundant microbes included five phyla (*Firmicutes*, *Planctomycetes*, *Proteobacteria*, *GN02*, and *Bacteroidetes*), eight family (*Bacillaceae*, *Vibrionaceae*, *Comamonadaceae*, *Moraxellaceae*, *Shewanellaceae*, *Enterobacteriaceae*, *Oceanospirillaceae*, and *Pseudomonadaceae*) and 11 genus (*Tenacibaculum, Photobacterium, Acinetobacter, Aliivibrio, Crocinitomix, Enterovibrio, Pseudoruegeria, Chryseobacterium, Delftia, Klebsiella,* and *Plesiomonas*) were found to correlate with seasonal variations (Fig. 2 and Fig. S2). Although the captive group exhibited a stronger correlation with seasonal variations, both the wild and captive groups showed a similar trend from spring to summer. These evidences indicated that the trend in the abundance of specific bacteria in both wild and captive *E. akaara* are affected by seasonal variation, similar to the yearlong study of changes in predominance of *Lactococcus lactis* in *Cyprinus carpio* [52] and seasonal variations among bacterial species, including *Escherichia coli* and *Klebsiella* spp., in *Ictalurus Punctatus* [53]*.* In contrast, a remarkably stable community composition of gut microbiota was observed throughout the year in farmed Atlantic salmon [50], indicating variable composition and community structure as affected by seasonal variations.

Our previous research highlighted the significant impact of water environmental factors and microbiota on fish gut microbiota, however, source tracking analysis revealed that the effect of water environmental microbiota was relatively small, especially on the midgut, hindgut, and contents [35]. Therefore, given the diverse environmental factors in aquatic ecosystems, it is crucial to assess the physicochemical properties of water when investigating the microbial composition of marine fish [8]. Our study revealed significant correlations between these 12 environmental factors and the microbial communities of both captive and wild *E. akaara* throughout all four seasons, such as SiO_4_^2−^, NO_3_^−^, NO_2_^−^ and WT. Nevertheless, varying significant correlations were observed in different seasons for both captive and wild *E. akaara*. These alterations in microbial communities could be elucidated as follows. First, changes in WT lead to a cascade of responses in fish, including homeostatic adjustments, metabolic changes, and alterations in gut microbiota [54, 55]. WT fluctuations may drive changes in environmental factors, subsequently influencing the fish gut microbiota. For instance, only the winter group in both captive and wild *E. akaara* exhibited significant correlation with WT (Fig. 3). These findings further emphasize the potential significance of physicochemical factors in shaping fish microbial communities throughout all four seasons.

In various vertebrates, including deer mice, the gut microbiome α-diversity is higher in natural environments compared to captive deer mice [16], as has been observed in humans [56, 57] and animals [58, 59]. In fish, our study further confirmed this pattern, finding higher levels of α-diversity in wild *E. akaara* compared to captive *E. akaara,* consistent with previous studies (Fig. S11). In comparison to captive animals, wild conspecifics are exposed to a broader array of microbial meta-communities through environmental sources, such as greater range of motion, seasonality, social interactions, and a wide range of diets. This exposure contributes to increased diversity of the gut microbiota [14, 60–62]. However, in certain cases, conspecifics have not exhibited variations in gut α-diversity between wild and captive environments, as observed in lizards [63], even-toed ungulates (*Cetartiodactyla*) and two myrmecophagus species [59]. Comprehending the factors contributing to these discordant outcomes can facilitate further research into the gut microbiota of both wild and captive populations.

The network analysis was conducted to comprehensively understand the interactions, composition and assembly rules that mirror the ecological processes of fish microbial community [64]. Our study had identified noteworthy distinctions in the networks of *E. akaara* between the captive and wild groups, indicating its distribution was non-random, which corresponded with the topological characteristics of a small-world network and an intrinsic modular architecture (Fig. 4a). Interestingly, the network complexity of the captive group was significantly higher than the wild group. We also observed the presence of more positively correlated bacteria in captive *E. akaara,* thus promoting cooperation and complementarity. This finding is noteworthy, especially considering that wild *E. akaara* exhibited higher levels of gut microbiome α-diversity. The proportion of positive interactions was notably greater within the gut microbiota of both wild and captive *E. akaara*. When the value of the modularity index greater than 0.4, a highly modularized structure is defined by the clustering of multiple interacting species, which contributes to the stability of interaction networks and facilitating the microbial community's adaptation to environmental fluctuations [65–67]. The presence of non-random co-occurrence patterns had offered novel insights into the impact of correlations between wild and captive populations on the assembly of *E. akaara* microbial community.

The gut microbiota of wild and captive *E. akaara* was predominantly composed of *Proteobacteria*, with wild *E. akaara* exhibiting a significantly higher relative abundance of *Proteobacteria* compared to their captive counterparts (Fig. S6). Both wild and captive *E. akaara* exhibited lower abundance of *Firmicutes* and *Bacteroidetes* compared to *Proteobacteria*. The relative abundance of *Firmicutes* was notably higher in the captive group, while *Bacteroidetes* exhibited significantly greater prevalence in the wild group. Previous studies on marine fish, both wild and captive, have consistently shown that the gut microbiota is primarily composed of *Proteobacteria* [35, 51]. In addition, fluctuations in *Firmicutes* and *Bacteroidetes* abundance within the gut microbiota of wild and captive *E. akaara* may be associated with variations in digestive efficiency. This might be of utmost importance for wild fish, as they face threats in their natural habitat and must optimize energy extraction from their diet, unlike captive environments [16, 47]. Differences in the gut microbiota of wild and captive *E. akaara* were also influenced by alterations in the relative abundance of specific microbial families. *Vibrionaceae*, *Clostridiaceae*, *Flavobacteriaceae* and *Rhodobacteraceae* were present in the gut microbiota of wild *E. akaara,* and have been previously associated with potential opportunistic pathogens [68–70], while *Bacillaceae*, *Moraxellaceae* and *Enterobacteriaceae* were found to be associated with microbial communities in captive *E. akaara* (Fig. 4c), and their presence may be affected by the nutrition composition of the regular diet [35]

Prior research has reported that the gut microbiome of vertebrates, including humans, chickens and fish, plays a pivotal role in host health, and its composition and function varying by location [71–74]. *Bacteroides spp.* in the human colon possess the capability to absorb and metabolize fatty acids and simple carbohydrates from food [68, 73]. In fish, microbial communities are evenly distributed and well-adapted to the specific gut environment, which related to their potential functions and survival requirements [35]. Notably, changes in the micro-biogeography of the gut microbiota during illness are also of significance [70]. In this study, we combined data from different time scales (across seasons) and culture environments (wild and captive) to provide a comprehensive and reliable description of distribution characteristics of the gut microbiota in *E. akaara*. It was found that the gut microbiota distribution of most *E. akaara* showed a pattern from the foregut to the hindgut, such as a decreasing trend of *Firmicutes* at the phylum level, as well as *Bacillaceae*, and *Rhodobacteraceae* at the family level (Fig. 5a and Fig. 6b). In contrast, an upward trend of *Proteobacteria* was observed at the phylum level from the foregut to the hindgut, as well as *Vibrionaceae*, *Moraxellaceae* and *Pseudomonadaceae* at the family level. These findings coincided with prior studies on the gut microbiota of eight marine fish species in Dongshan city and the gut microbiota captive *E. akaara* in five cities (including Dongshan, Quanzhou, Putian, Fuzhou, Ningde), which highlighted the distribution patterns of *Firmicutes* and *Proteobacteria* at phylum level, as well as *Vibrionaceae* and *Bacillaceae* [35]. In addition, we observed a substantial difference in the content microbiota composition compared to the other segments, with the dominant taxa being *Proteobacteria* and *Vibrionaceae*, similar to the results on the microbiota of *Epinephelus coioides, Siganus fuscescens, Pagrus major, Lateolabrax japonicas, Acanthopagrus schlegelii, Gadus morhua*, *Poecilia reticulate* and *Scophthalmus maximus* [35, 65, 75, 76]. However, it differed from other vertebrate species, such as, humans and chickens, whose fecal communities exhibited higher abundance of *Firmicutes* and *Bacteroidetes* [71, 73]. Notably, despite the relatively high standard deviations observed at each gut parts, these results comprehensively summarized the impact factors related to time scales and different survival cultures on the gut microbiota of *E. akaara*.

## Conclusions

In summary, this study presents the relatively more comprehensive description of the gut microbiota in *E. akaara* in multiple dimensions, including time scales and different ecological conditions, providing valuable insights into *E. akaara* as a model for mariculture research. Although captive and wild *E. akaara* showed overall similarity, the abundance of certain taxa such as *Bacillaceae*, *Moraxellaceae* and *Enterobacteriaceae* increased in the captive environment, in contrast, *Vibrionaceae*, *Clostridiaceae*, *Flavobacteriaceae* and *Rhodobacteraceae* are prominent active taxa in the wild environment, indicating strong host selection. Environmental factors, such as WT, can have a substantial impact on the composition of microbial communities in *E. akaara* across all four seasons. Notably, although certain core microbes exhibited consistent distribution patterns in both captive and wild group, there are also some notable variations in α-diversity across four seasons, displaying clear regularity. Combined with the database of wild and captive *E. akaara* in four seasons, we further characterized the common community composition and distribution. Our findings highlight the importance for researchers to exercise caution and take an integrative approach when analyzing the gut microbiota of fish, taking into account factors such as time scales and different survival cultures simultaneously.

## Materials and methods

### Sample collection and DNA extraction

During 2018 and 2019, we collected a total of 72 fish (9 fish per group) and 72 water samples (from a depth of 0.5 m below the water surface, with 9 parallel samples per group) in Donshan City, Fujian Province, China, over four seasons (from spring to winter). Detailed site information and locations can be found in Fig. 1a and Table S5. The captive *E. akaara* were all collected from Tengsheng Breeding Company (Dongshan, Fujian province, China), and the diet of captive *E. akaara* feeding was commercially available formulated diet for grouper (Fuzhou Haima Feed Co. Ltd, China). The composition of the diet was as follows: crude protein ≥ 44%, crude fat ≥ 9%, lysine ≥ 2.3%, crude ash ≤ 18%, crude fiber ≤ 6%, moisture ≤ 12%, total phosphorus ≥ 1%, Calcium 0.8%-4.0%, and sodium chloride 0.3%-3.5%. The wild *E. akaara* were captured from Dongshan offshore angling marine waters. We filtered 1 L of water for 16S rRNA sequencing using a 0.2-mm pore size polycarbonate membrane (Millipore, Massachusetts, USA). After anesthesia with ethyl 3-aminobenzoate methanesulfonate salt analytical standard (0.1 g/L, 2 min immersion, MS-222, Sigma-Aldrich, USA), the gut was carefully dissected with sterile instruments, and divided evenly into the foregut, midgut, and hindgut. Subsequently, the contents of each section were gently squeezed out and meticulously collected into sterile cryovials to ensure complete evacuation of the gut's contents, as described in our previous study [35]. Each intestinal segment from three parallel individual fish constituted an independent sample, with three samples per gut segment. These fish were approximately 1 year old, weighing around 200 g. A trained research technician from the institute consistently performed all treatments in a uniform manner throughout the experiment.

The microbial genome extraction for each sample followed our prior study [35], employing the QIAamp DNA Stool Mini Kit (Qiagen, Hilden, Germany), ensuring adherence to subsequent sequencing requirements.

### 16S rRNA sequencing of the gut microbiome

PCR-amplified V4 region was selected for sequencing, utilizing the Illumina MiSeq 2000 Next Generation system. Sequencing was performed at Gene Denovo Biological Technology Co. Ltd. (Guangzhou, China). The primers, experimental conditions, procedures, and related kits employed in this study were consistent with the descriptions provided in our prior study [35], culminating in the formation of the ultimate amplicon library.

### Sequences data processing

Data quality control (QC) and analysis were conducted using the Quantitative Insights Into Microbial Ecology (v1.8.0) pipeline [77]. Subsequently, high‐quality data were integrated with tags through the FLASH software. Utilizing USEARCH (v9.0), the tags were clustered into operational taxonomic units (OTUs) with a 97% identity threshold. Representative OTU sequences were acquired and subjected to taxonomic annotations, achieved through the Greengene database (v.13.8) [78] and RDP Classifier (v2.2) software with a set confidence threshold of 0.5. To account for variations in sequence depths across samples, all datasets were standardized by subsampling to 6,000 reads per sample. Finally, the OTU abundance for each sample and a six-level taxonomic classification spanning from phylum to species were determined.

### Physicochemical factors analysis

On-site measurements of environmental variables, encompassing pH, salinity, water temperature (WT), and dissolved oxygen (DO), were conducted using the Combo Water Quality Meter (86,031, AZ Instrument Corp, China).. Nutrient samples, such as NO_3_^−^, NO_2_^−^, SiO_4_^2−^, AN (Ammonia nitrogen), PO_4_^3−^, NPOC (particulate organic carbon) and chlorophyll, were all detected using the methods and instruments previously described in our study [35].

### Comparison of gut communities and bioinformatics analysis

The qualified OTU data were employed to compute α-diversity metrics through the QIIME software package [77]. Subsequently, a one-way analysis of variance (ANOVA) with Bonferroni's post hoc test was performed using SPSS software (SPSS, Chicago, IL, USA). Bray–Curtis dissimilarities were employed as measures of β-diversity, followed by principal coordinate analysis (PCoA) carried out using the QIIME software package and vegan package. The mantel test was conducted to ascertain the correlation between microbial communities and the environmental variables of both captive and wild *E. akaara* across various seasons, employing the ggcor R package, using the Euclidean distance metric to compute the dissimilarities between environmental samples, while employing the Bray–Curtis distance metric to quantify dissimilarities within the microbial community OTU data matrix. And the gut microbiota of each *E. akaara* across different seasons comprises sequenced OTUs obtained from 12 samples, with 3 samples collected from the foregut, 3 from the midgut, 3 from the hindgut, and 3 from the content. To evaluate the influence of WT, NO_2_^−^, NO_3_^−^ and SO_4_^2−^ on the structure of networked communities between captive and wild groups, a Redundancy Analysis (RDA) model was employed. Subsequently, VPA was conducted to delineate the respective contributions of these variables to the overall variations observed in the networked communities using the WGCNA packages in R [79].

We constructed a co-occurrence network for microbial communities in *E. akaara* with distinct survival characteristics. To depict the associations within the network, we generated a correlation matrix through the computation of pairwise Spearman's rank correlations [37]. The nodes in the reconstructed network represented bacterial taxa (OTUs), and the edges represented highly significant correlations between nodes. To characterize the complex pattern of interrelationships among bacterial OTUs, the topological features of the networks were calculated as follows: average path length (APL), graph density, network diameter, average clustering coefficient (avgCC), average degree (avgK), and modularity (M). We conducted network analysis utilizing the igraph, vegan, and Hmisc packages within the R software [37]. Subsequently, the correlation networks were visualized through Gephi software. Species classification trees were generated with the metacoder packages in R [80]. The robustness of a network is defined and analyzed using the WGCNA packages in R, following the method described previously [79]. We employed a modified Raup–Crick null model to quantify the extent to which environmental variables and seasons contribute to the variation in microbial community structure across different gut segments [81].

All analyses were performed using R (version 3.5.1, R Development Core Team), unless specified otherwise.

### Supplementary Information

Supplementary Material 1.

Supplementary Material 2.

## Data Availability

The datasets generated during the current study are available in the NCBI repository, accession number PRJNA1014228.
