# Supplementary material for: Impact of captivity and natural habitats on gut microbiome in Epinephelus akaara across seasons
Source: BMC Microbiol. 2024 Jul 3;24:239. doi: 10.1186/s12866-024-03398-y (PMC11221007; doi:10.1186/s12866-024-03398-y)
Supplement: Supplementary file 2 — Supplementary Material 2. [file 12866_2024_3398_MOESM2_ESM.docx]

**Impact of captivity and natural habitats on gut microbiome in *Epinephelus akaara* across seasons**

Hang Sun^1^, Fangyi Chen^1,2,3^, Wenbin Zheng^1^, Yixin Huang^1^, Hui Peng^1,2,3^, Hua Hao^1,2,3^and Ke-Jian Wang^1,2,3*^

^1^ State Key Laboratory of Marine Environmental Science, College of Ocean and Earth Sciences, Xiamen University, Xiamen, Fujian, China

^2^ State-Province Joint Engineering Laboratory of Marine Bioproducts and Technology, College of Ocean and Earth Sciences, Xiamen University, Xiamen, Fujian, China

^3^ Fujian Innovation Research Institute for Marine Biological Antimicrobial Peptide Industrial Technology, College of Ocean and Earth Sciences, Xiamen University, Xiamen, Fujian, China

^*^ Corresponding autor.

Ke-Jian Wang, E-mail address: wkjian@xmu.edu.cn

Supplementary Figures


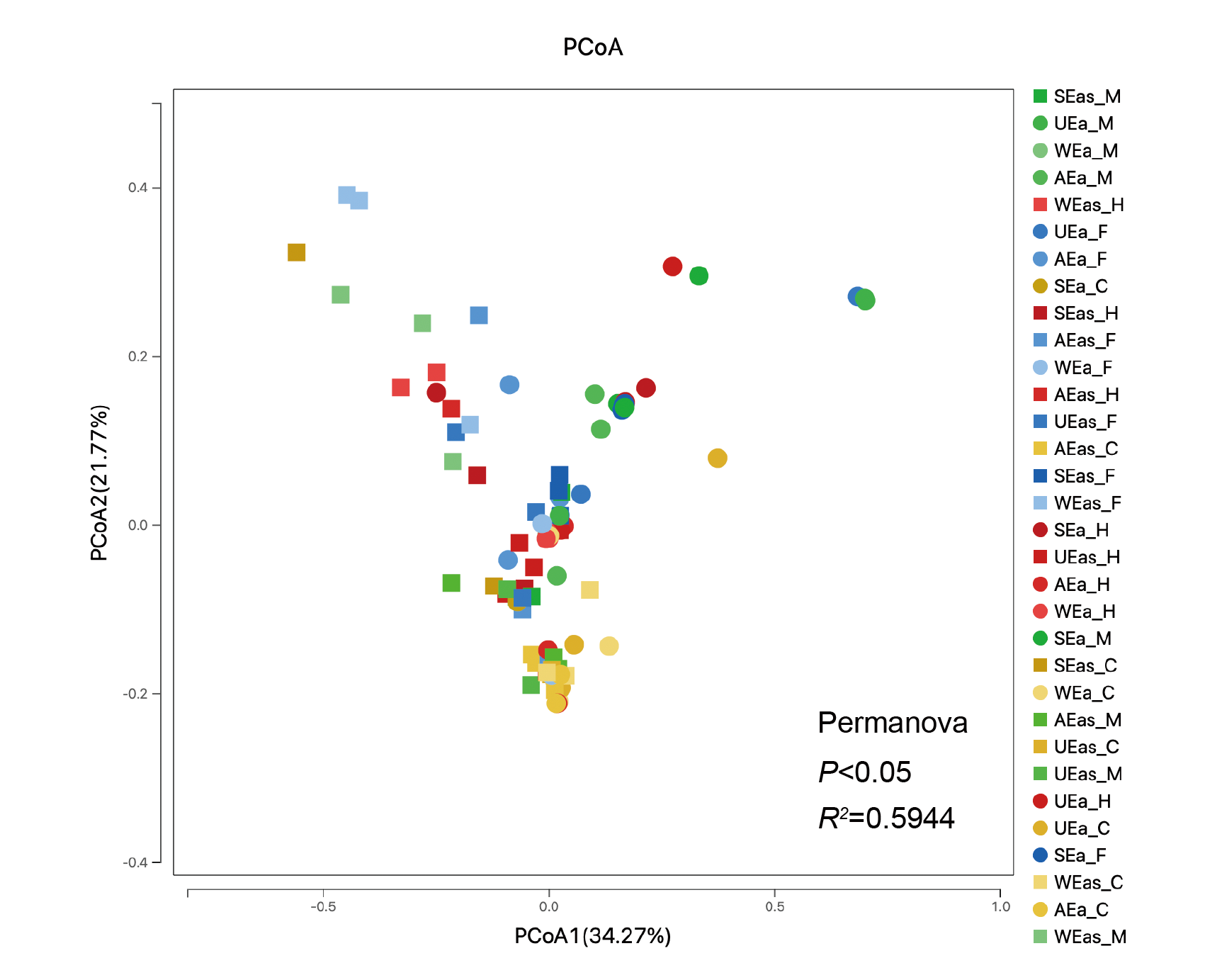


Fig. S1. Principal Coordinate Analysis (PCoA) plot illustrating the variations among gut parts across four seasons, constructed utilizing Operational Taxonomic Units (OTU) metrics derived from Bray-Curtis dissimilarity measurements. Each data point on the plot corresponds to a specific sample. The dissimilarities were statistically evaluated using PERMANOVA, with significance determined at a threshold of *P* < 0.05.


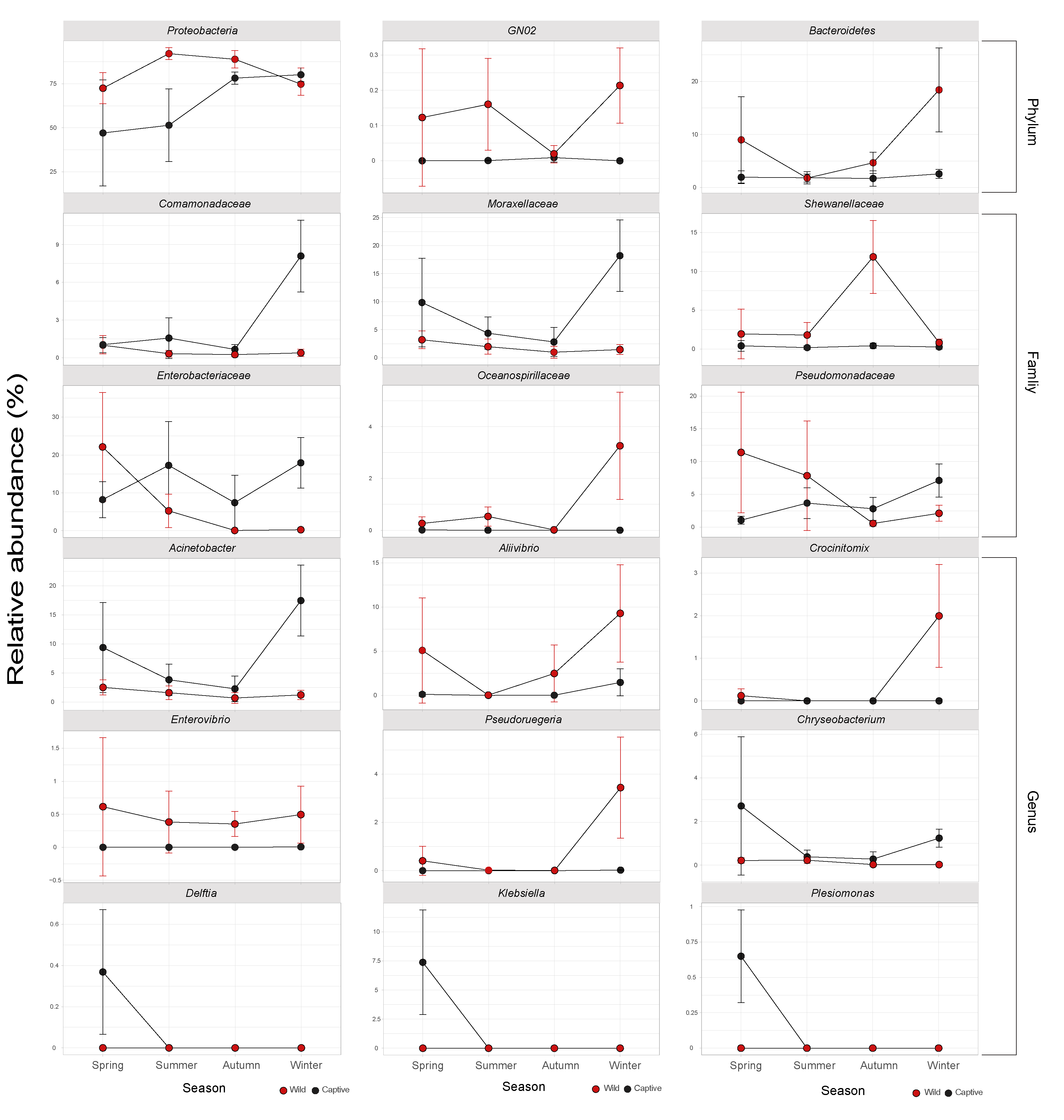


Fig. S2. The relative abundance of specificity patterns affected by seasonal variations at phylum, family and genus level. At phylum level, Bacteroidetes showed a rising trend after three seasons falling from spring to winter both in the captive and wild group. While *Proteobacteria* showed a decreasing trend from summer to spring and the abundance of GN02 decline sharply in autumn. At family level, the significant divergence of *Enterobacteriaceae*, *Pseudomonadaceae* and *Moraxellaceae* between wild and captive *E. akaara* occurred in spring and winter, when *Comamonadaceae* and *Oceanospirillaceae* showed significant difference in winter. At genus level, *Acinetobacter*, *Aliivibrio*, *Crocinitomix*, *Enterovibrio*, *Pseudoruegeria* and *Chryseobacterium* showed similar trends to Bacteroidetes with different degrees. While the significant divergence of *Delftia*, *Klebsiella* and *Plesiomonas* between wild and captive *E. akaara* occurred in spring. These finding further indicate the gut microbial communities of *E. akaara* may be affected by seasonal variation.


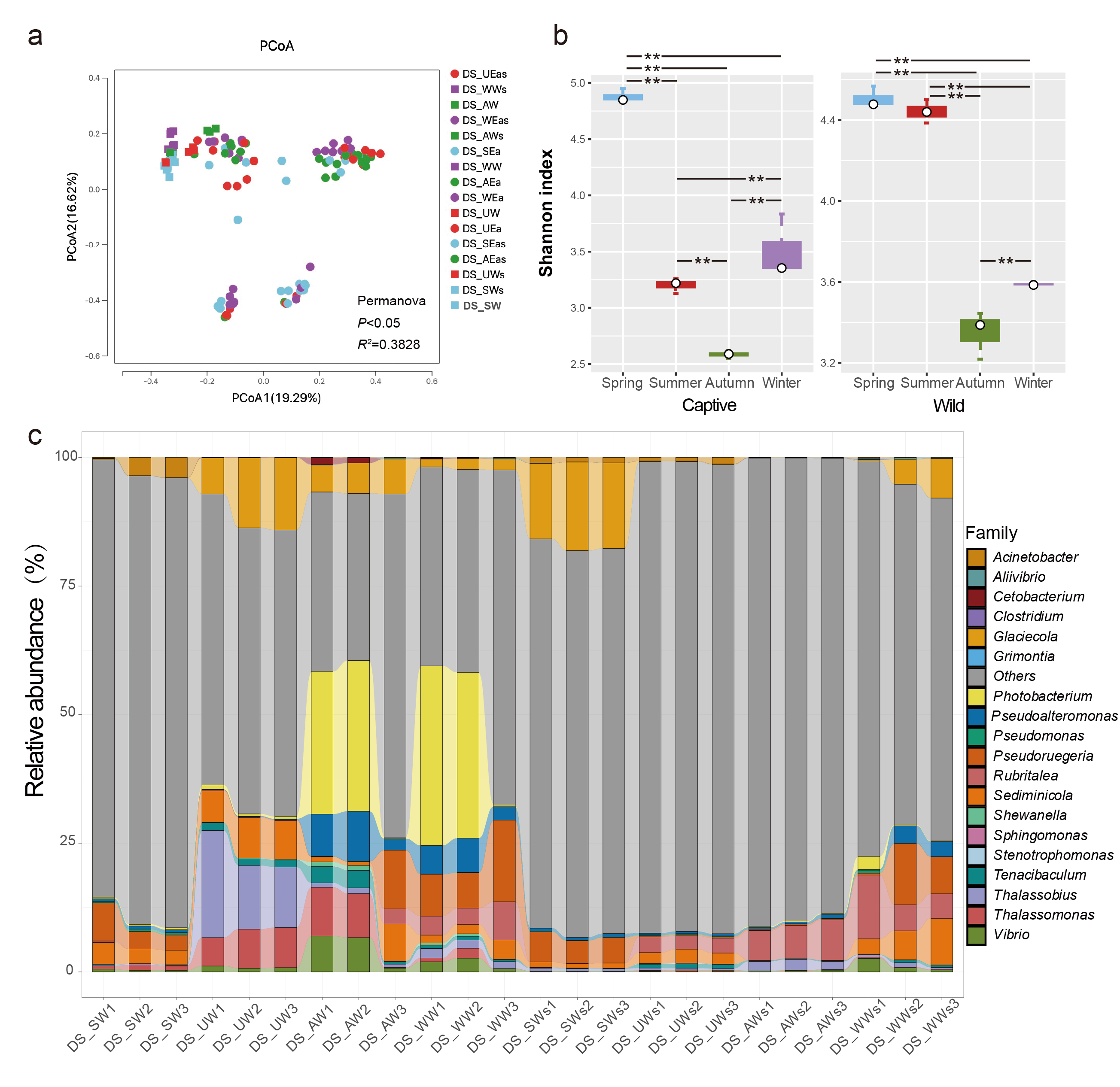


Fig. S3. Seasonal Dynamics of Microbial Community Composition in Aquatic Environments. (a), Principal coordinate analysis plot generated using OTU metrics based on the Bray-Curtis dissimilarities. Each point represents a sample. Differences were assessed by PERMANOVA and significance was established at *P* < 0.05. (b), ɑ-Diversity comparison based on the Shannon diversity index in different seasons between captive and wild group (**P* < 0.05, ** *P* < 0.01). (c), The relative abundance of the microbial communities at the genus level found in water environment of different seasons. Only the dominant microbial family with top 20 of the sites are plotted.


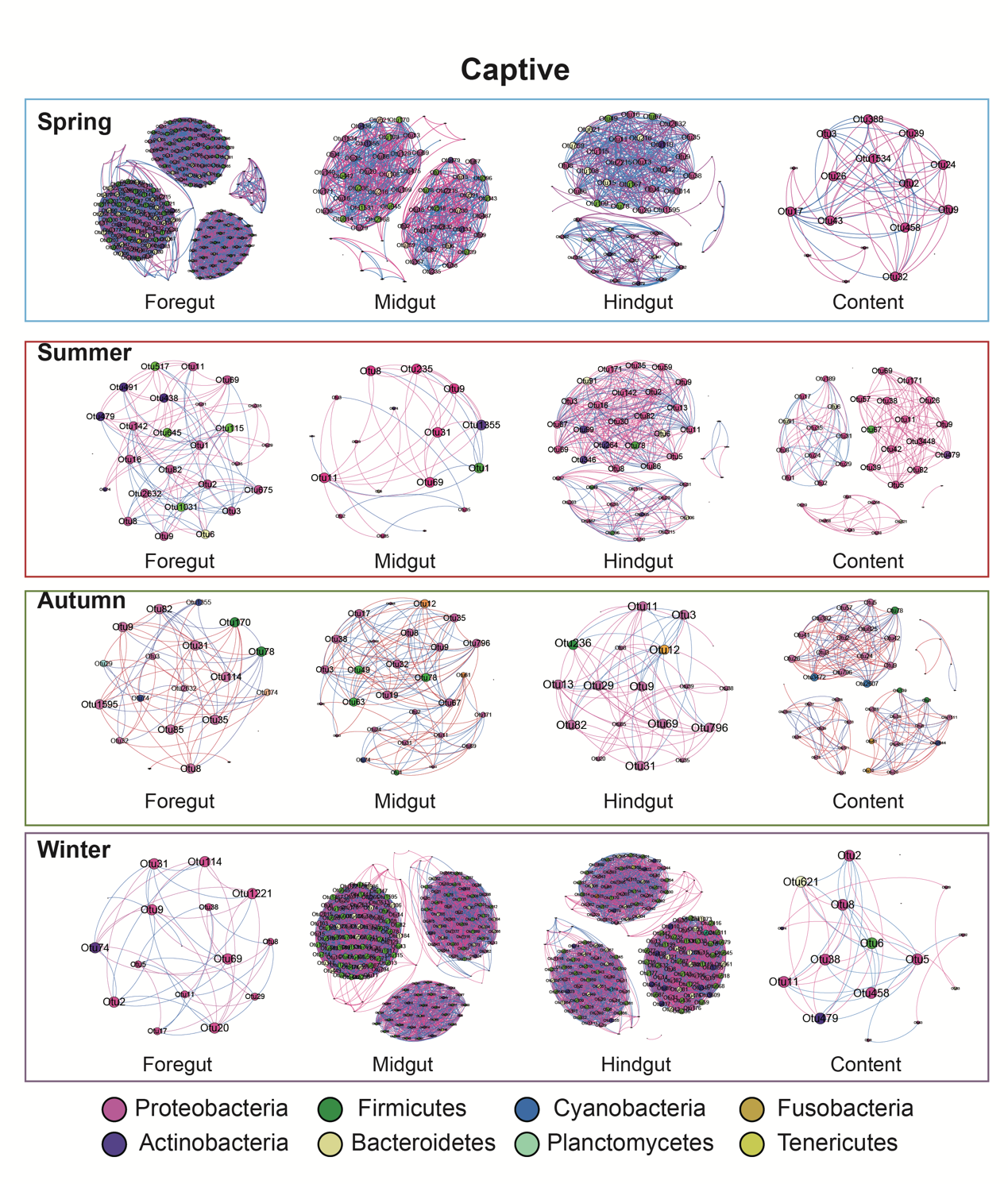


Fig. S4. The networks of co-occurring bacterial OTUs in different gut parts of captive *E. akaara*, based on correlation analysis. The co-occurring networks are colored by phylum. A blue edge indicates a positive interaction between two individual nodes, while a red edge indicates a negative interaction. And the robustness measured as the proportion of taxa remained with 50% of the taxa randomly removed from each of the empirical MENs.


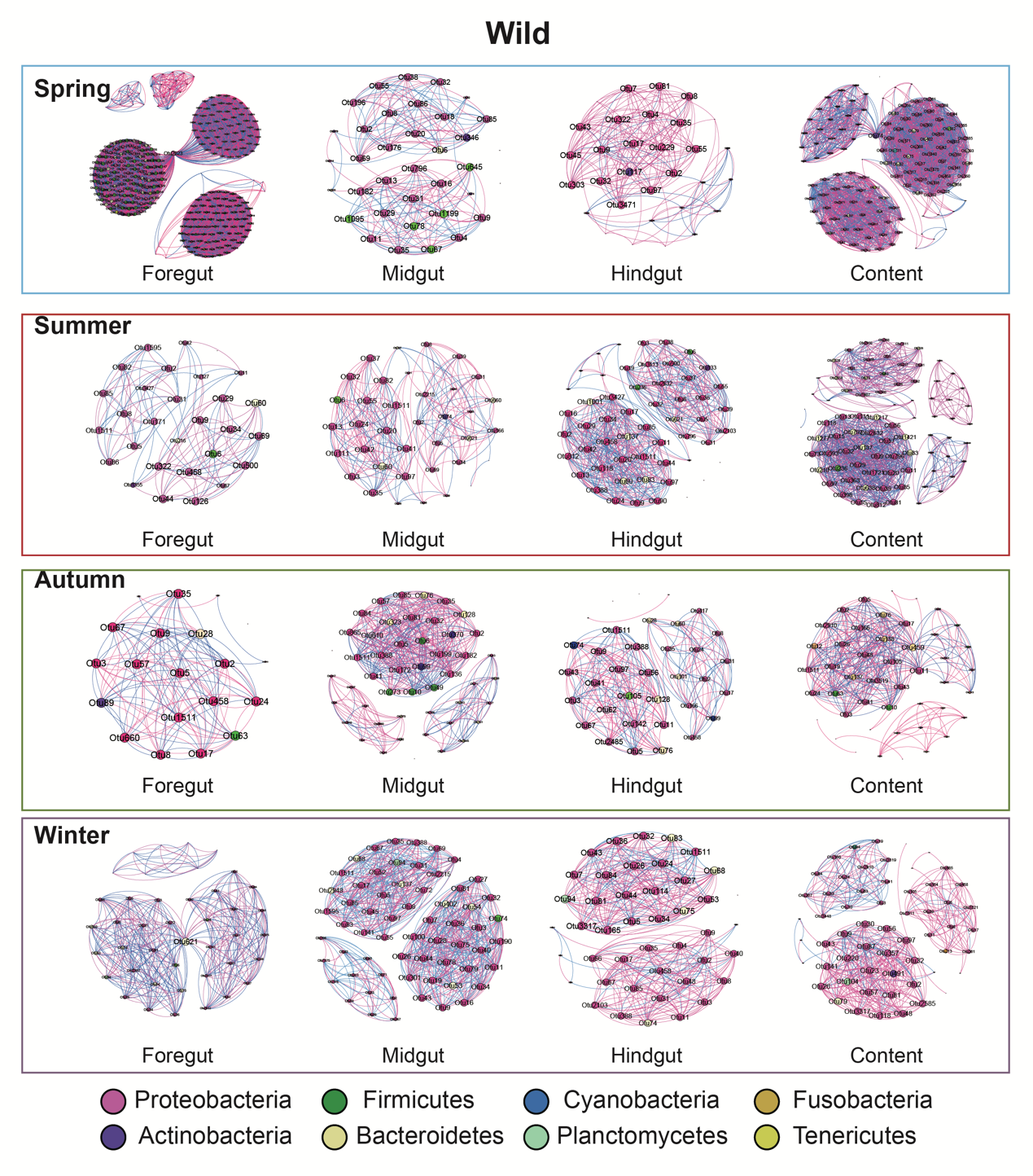


Fig. S5. The networks of co-occurring bacterial OTUs in different gut parts of wild *E. akaara*, based on correlation analysis. The co-occurring networks are colored by phylum. A blue edge indicates a positive interaction between two individual nodes, while a red edge indicates a negative interaction. And the robustness measured as the proportion of taxa remained with 50% of the taxa randomly removed from each of the empirical MENs.


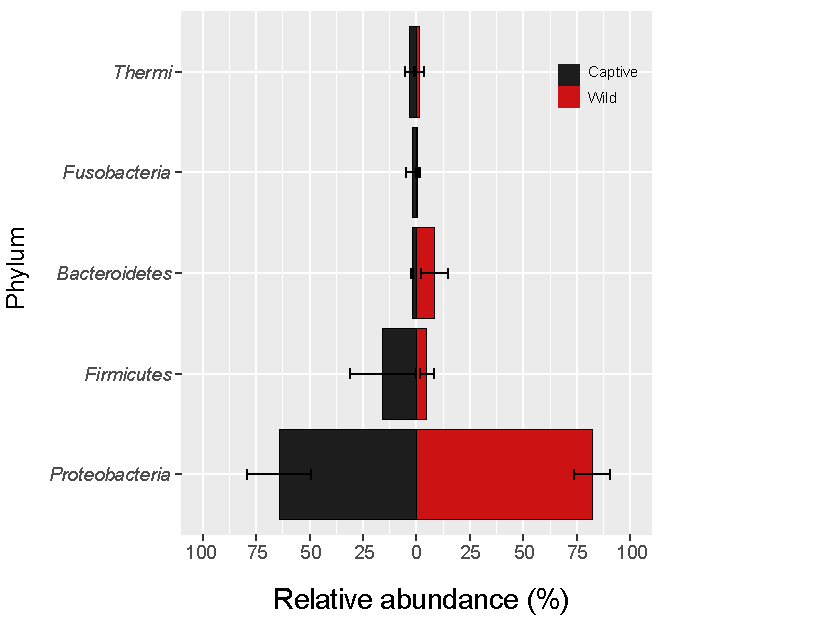


Fig. S6. Side-by-side comparison of the mean relative abundance of phylum which made up at least >1% of the total gut microbiome community, within at least one individual, between captive and wild *E. akaara.* Significant differences occurred between *Proteobacteria*, *Firmicutes*, *Bacteroidetes* and *Fusobacteria*. These finding further indicate the divergence of the composition of gut microbiota between captive and wild *E. akaara.*


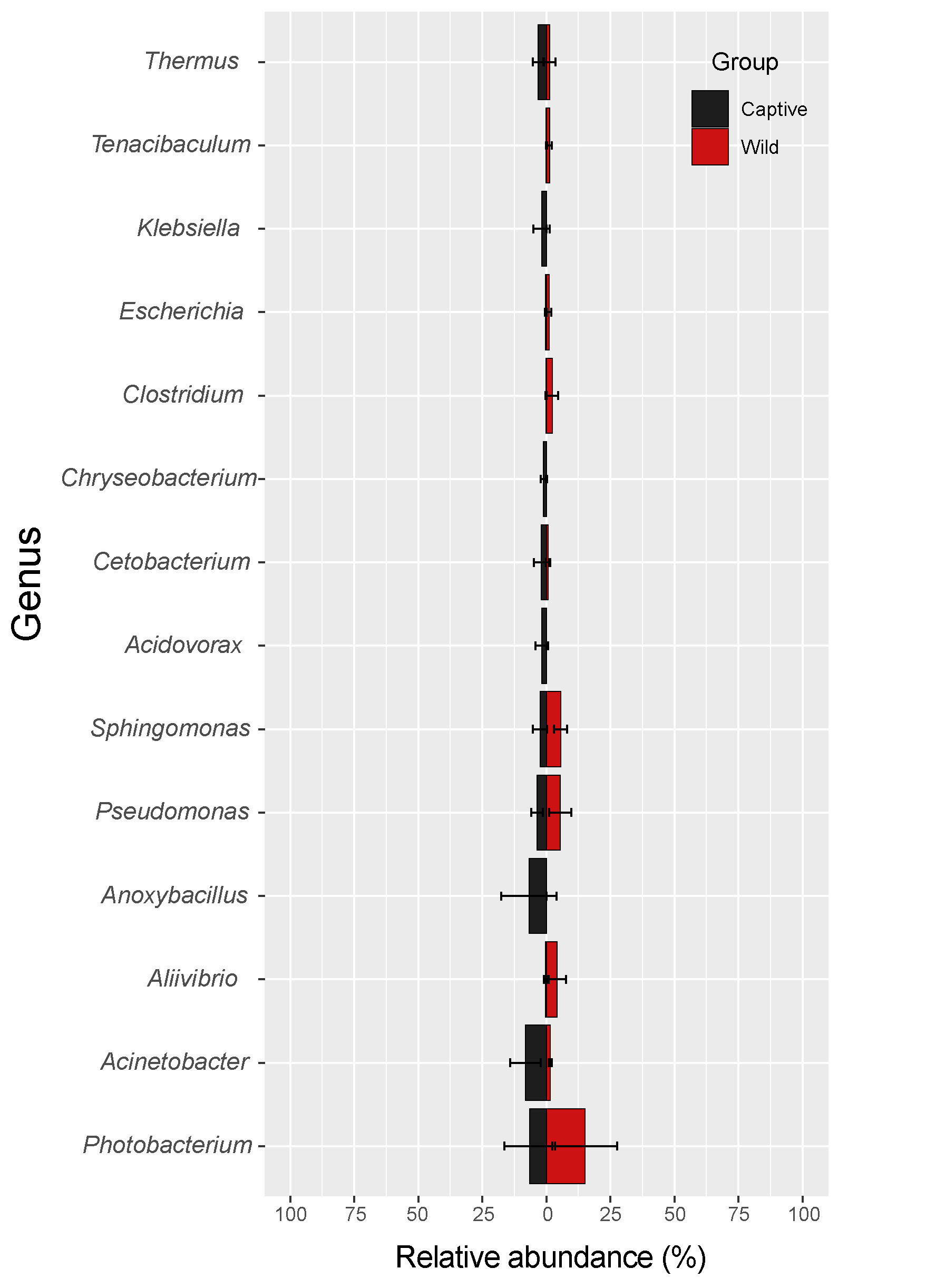


Fig. S7. Side-by-side comparison of the mean relative abundance of genus which made up at least >1% of the total gut microbiome community, within at least one individual, between captive and wild *E. akaara.* Significant differences occurred between *Photobacterium*, *Acinetobacter*, *Aliivibrio*, *Anoxybacillus*, *Pseudomonas*, *Sphingomonas* and *Clostridium*. These finding further indicate the divergence of the composition of gut microbiota between captive and wild *E. akaara.*


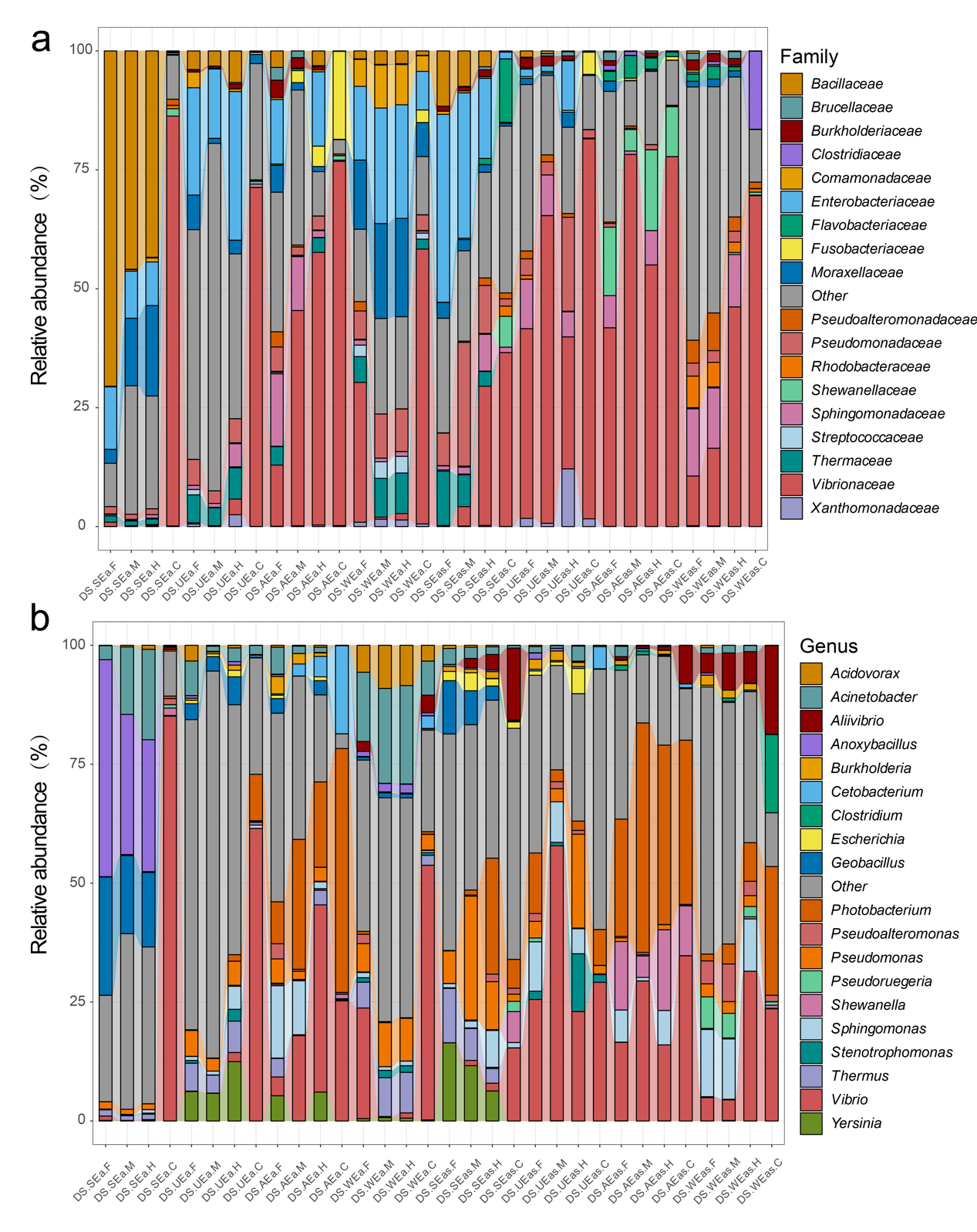


Fig. S8. (a), The relative abundance of the microbial communities at the family level found in different gut compartments (foregut, midgut, hindgut and content). Each group represents 9 fish individuals with a parallel sample mixed by three individuals. Only the dominant microbial family with top 20 of the sites are plotted. (b), The relative abundance of the microbial communities at the genus level found in different gut compartments (foregut, midgut, hindgut and content). Each group represents 9 fish individuals with a parallel sample mixed by three individuals. Only the dominant microbial family with top 20 of the sites are plotted.


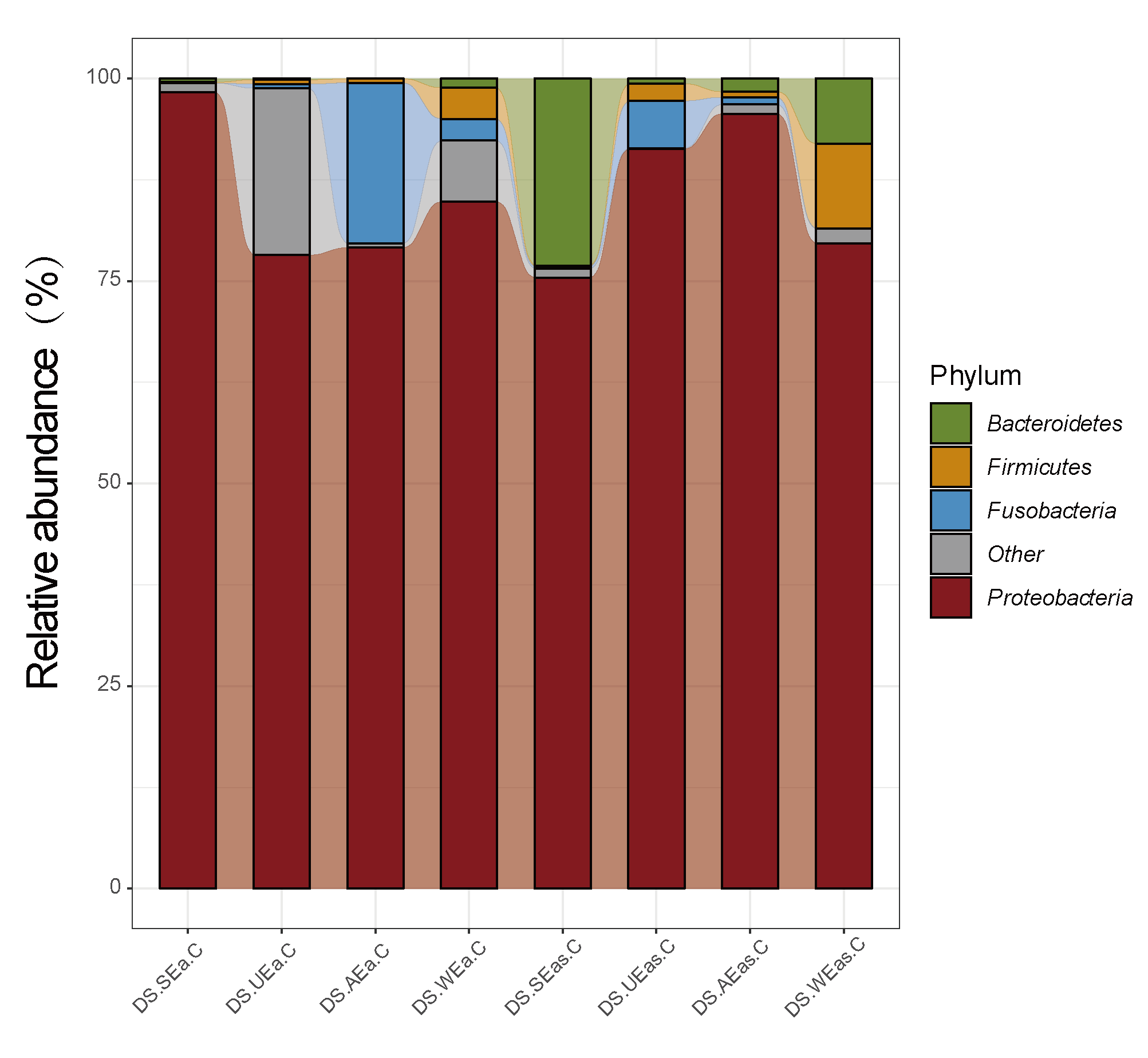


Fig. S9. The relative abundance of the microbial communities at the Phylum level found in content. Each group represents 9 fish individuals with a parallel sample mixed by three individuals.


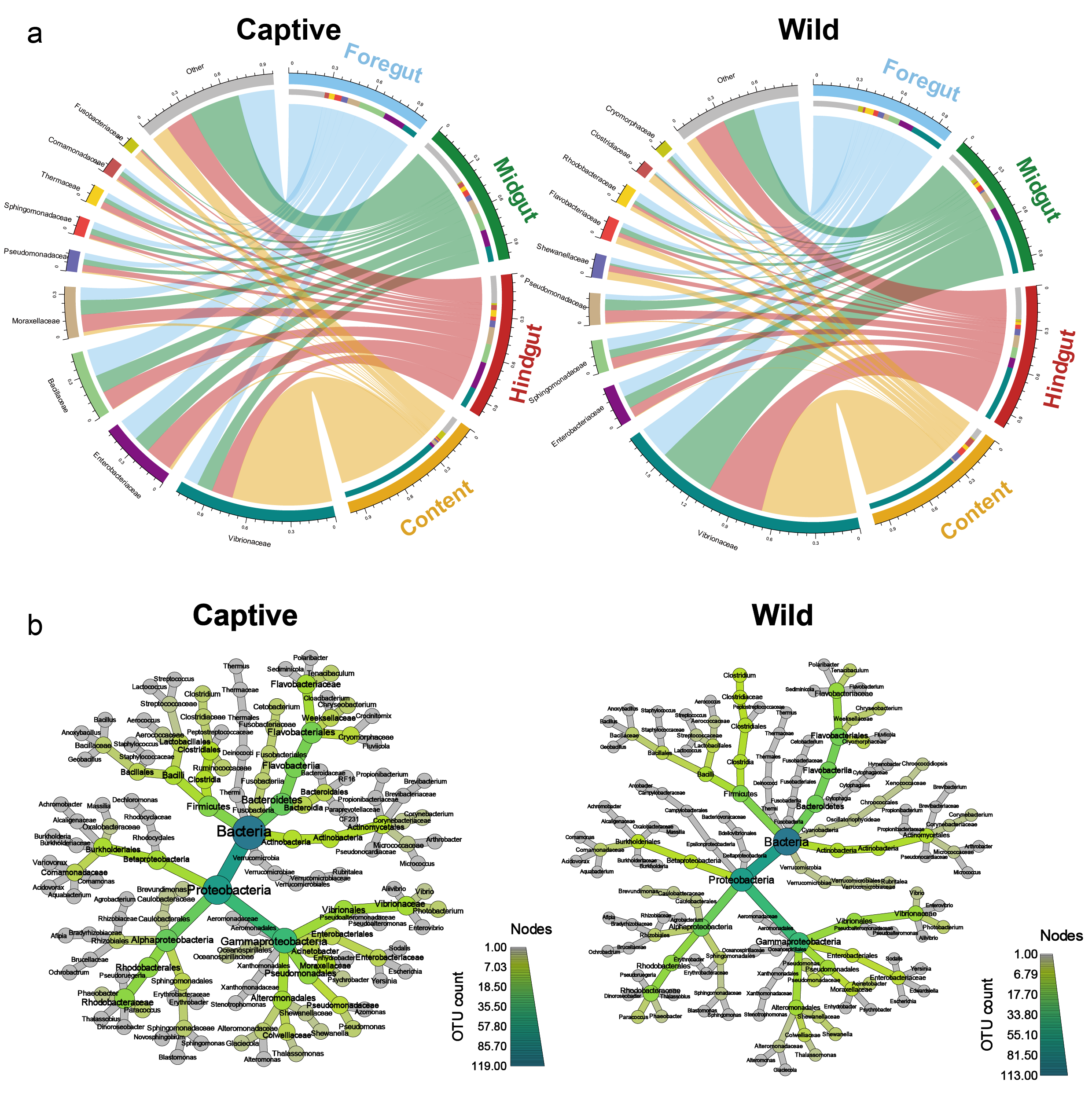


Fig. S10. Common distribution characteristics and composition of microbial communities in different gut fragments between captive and wild *E. akaara.* (a) Relative abundance of the top 10 family in samples of different gut compartments (foregut, midgut and hindgut) between captive and wild group. (c) The species classification tree displayed the mean proportion of bacterial components between captive and wild *E. akaara*. Nodes represent each taxonomic rank from kingdom (bacteria, center) to genus (tips of each branch). Node and edge (branch) width indicates the mean proportion of that taxon in samples belonging to that group. Size of nodes corresponds to the number of taxa and color intensity corresponds to proportions relative to bacterial samples overall. Only genus detected at ≥0.03 mean proportion are displayed.


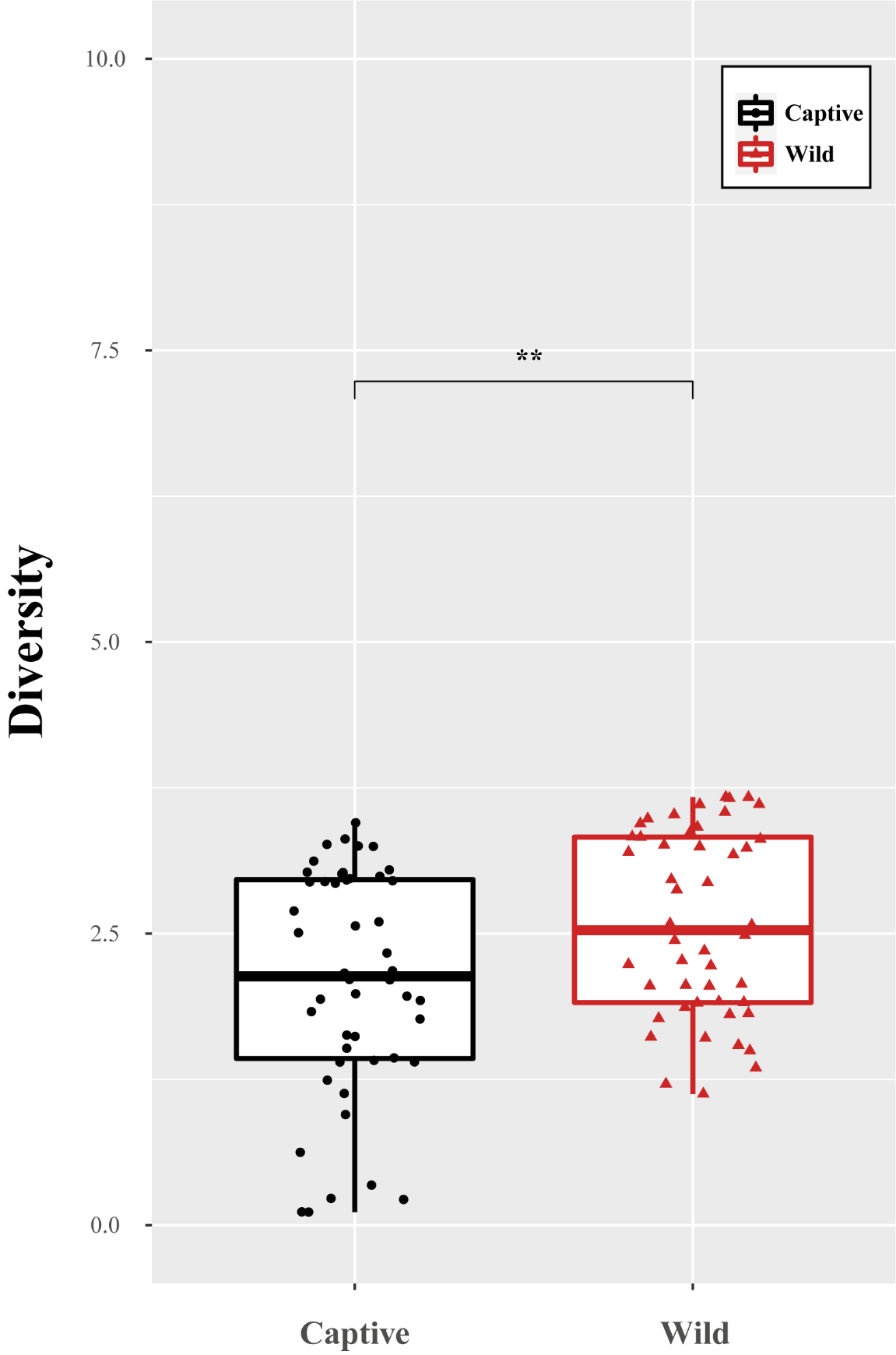


Fig. S11. ɑ-diversity comparison based on the Shannon diversity index between captive and wild *E. akaara* using ANOVA to determine significant differences (** *P* < 0.05, ns *P* > 0.05) In data shown as a combination of dot plots and box plots. The ɑ-diversity of wild group showed significant higher diversity compared to captive group.
